# Evaluating blended learning effectiveness: an empirical study from undergraduates’ perspectives using structural equation modeling

**DOI:** 10.3389/fpsyg.2023.1059282

**Published:** 2023-05-18

**Authors:** Xiaotian Han

**Affiliations:** Department of Elementary School Education, School of Primary Education, Shanghai Normal University Tianhua College, Shanghai, China

**Keywords:** blended learning effectiveness, measurement, undergraduates, student learning outcomes, structural equation modeling

## Abstract

Following the global COVID-19 outbreak, blended learning (BL) has received increasing attention from educators. The purpose of this study was: (a) to develop a measurement to evaluate the effectiveness of blended learning for undergraduates; and (b) to explore the potential association between effectiveness with blended learning and student learning outcomes. This research consisted of two stages. In Stage I, a measurement for evaluating undergraduates’ blended learning perceptions was developed. In Stage II, a non-experimental, correlational design was utilized to examine whether or not there is an association between blended learning effectiveness and student learning outcomes. SPSS 26.0 and AMOS 23.0 were utilized to implement factor analysis and structured equation modeling. The results of the study demonstrated: (1) The hypothesized factors (course overview, course objectives, assessments, 1148 class activities, course resources, and technology support) were aligned as a unified system in blended learning. (2) There was a positive relationship between the effectiveness of blended learning and student learning outcomes. Additional findings, explanations, and suggestions for future research were also discussed in the study.

## 1. Introduction

Following the global COVID-19 outbreak, blended learning (BL) has received increasing attention from educators. BL can be defined as an approach that combines face-to-face and online learning (Dos, 2014), which has become the default means of delivering educational content in the pandemic context worldwide due to its rich pedagogical practices, flexible approaches, and cost-effectiveness (Tamim, 2018; Lakhal et al., 2020). Moreover, empirical research has demonstrated that BL improves learners’ active learning strategies, multi-technology learning processes, and learner-centered learning experiences (Feng et al., 2018; Han and Ellis, 2021; Liu, 2021). Furthermore, students are increasingly requesting BL courses due to the inability to on-campus attendance (Brown et al., 2018). In addition, researchers have examined the positive effects of BL on engaging students, improving their academic performance and raising student satisfaction (Alducin-Ochoa and Vázquez-Martínez, 2016; Manwaring et al., 2017).

In China, the Ministry of Education has strongly supported educational informatization since 2012 by issuing a number of policies (the Ministry of Education, 2012). In 2016, China issued the Guiding Opinions of the Ministry of Education on Deepening the Educational and Teaching Reform of Colleges and Universities, emphasizing the promotion of the BL model in higher education. In 2017, the Ministry of Education listed BL as one of the trends in driving education reform in the New Media Alliance Horizon Report: 2017 Higher Education Edition. In 2018, Minister Chen Baosheng of the Ministry of Education proposed at the National Conference on Undergraduate Education in Colleges and Universities in the New Era to focus on promoting classroom revolution and new teaching models such as flipped classroom and BL approach. In 2020, the first batch of national BL courses was identified, which pushed the development of BL to the forefront of teaching reform. During the pandemic era in China, BL was implemented in all universities and colleges.

However, a number of researchers produced opposing results regarding the benefits of BL. Given the pre-requisites, resources, and attitudes of the students, BL model is suspected to be inapplicable to all courses, such as practicum courses (Boyle et al., 2003; Naffi et al., 2020). Moreover, it should be noted that students, teachers, and educational institutions may lack BL experience and therefore they are not sufficiently prepared (such as technology access) to implement BL methods or focus on the efficiency of BL initiatives (Xiao, 2016; Liliana, 2018; Adnan and Anwar, 2020). Another big concern is that BL practice is hard to evaluate because there are few standardized BL criteria (Yan and Chen, 2021; Zhang et al., 2022). In addition, a number of studies have concluded there was no significant contribution of BL in terms of student performance and test scores, compared to traditional learning environments (U.S. Department of Education, 2009). Therefore, it is extremely necessary to explore the essential elements of BL in higher education and examine the effect of BL on student academic achievement. This paper offers important insights for those attempting to implement BL in classroom practice to effectively support student needs in higher education.

The purpose of this research was: (1) To develop a measurement with key components to evaluate BL in undergraduates; (2) To explore the associations between perceptions of BL effectiveness and student learning outcomes (SLOs) in a higher education course using the developed measurement.

The significance of the current study was listed as follows: (1) The researchers noted that there have only a few studies have focused on the BL measurement in higher education and its effects on SLOs. Therefore, the current study results will add to the literature regarding BL measurement and its validity. (2) The Ministry of Education in China has an explicit goal the desire to update university teaching means and strategies in accordance with the demands of the twenty-first century. Therefore, the current study will contribute to the national goals of the Ministry of Education in China, enhance understanding of BL, and provide a theoretical framework and its applicability. (3) Faculty members in higher education who attempt to apply BL model in their instructions will be aware of the basic components of BL that contribute to SLOs.

## 2. Literature review

### 2.1. Definitions of BL

BL is referred to as “hybrid,” “flexible,” “mixed,” “flipped” or “inverted” learning. The BL concept was first proposed in the late 20 century against the backdrop of growing technological innovation (Keogh et al., 2017). The general definition of BL is that it integrates traditional face-to-face teaching with a web-based approach.

However, this description has been hotly debated by researchers in recent years. Oliver and Trigwell (2005) posited that BL may have different attributions in relation to various theories, meaning that the concept should be revised. Others attempted to clarify the significance of BL by classifying the proportion of online learning in BL and the different models that come under the BL umbrella. Allen and Seaman (2010) proposed that BL should include 30–70% online-in person learning (otherwise, it would be considered online learning (more than 70%) or traditional face-to-face learning (less than 30%)). In *The Handbook of Blended Learning* that edited by Bonk and Graham (2006) set out three categories of BL: web-enhanced learning, reduced face-time learning, and transforming blends. Web-enhanced learning pertains to the addition of extra online materials and learning experiences to traditional face-to-face instruction. Reduced face-time learning means to shift part of face-to-face lecture time to computer-mediated activities. Transforming blends mixes traditional face-to-face instruction with web-based interactions, through which students are able to actively construct their knowledge.

This study views BL as an instructional approach that provides both synchronous and asynchronous modes of delivery through which students construct their own understandings and interact with others in these settings, which is widely accepted by numerous researchers (Liliana, 2018; Bayyat et al., 2021). To phrase this in another way, this description emphasizes that learning has to be experienced by the learner.

### 2.2. Essential elements of BL

Previous studies, universities, and cooperation have discussed the essential components of online learning courses. Blackboard assesses online learning environments on four scales (course design, cooperation, assessment, and learner support) with 63 items. Quality Matters evaluated online learning according to the following categories: course overview, objectives, assessment, teaching resources, activities and cooperation, course technology, learner support, and practicability. Californian State Universities rated their criteria on a ten scale of 58 items, including learning evaluation, cooperation and activities, technology support, mobile technology, accessibility, and course reflection. New York State Universities evaluate BL under the following six sub scales: course overview, course design, and assignment, class activities, cooperation, and assessment. Due to the lack of criteria for BL, these standards have been considered in evaluating BL.

The present study utilized Biggs’ (1999) constructive alignment as the main theoretical framework to analyze BL courses. “Constructive means the idea that students construct meaning through relevant activities … and the alignment aspect refers to what the teachers do, which is to set up a learning environment” (Biggs, 1999, p. 13). Later, Biggs and Tang (2011) elaborated on the two terms — ‘constructive’ and ‘alignment’ originated from constructivist theory and curriculum theory, respectively, in the book Teaching for Quality Learning at University. Constructivism was regarded as “learners use their own activity to construct their knowledge as interpreted through their own exiting schemata.” The term “alignment” emphasized that the assessments set were relevant and conducive to the intended learning goals (Biggs and Tang, 2011, p. 97). According to Biggs’ statement, various critical components should be closely linked within the learning context, including learning objectives, teaching learning activities, and assessment tasks. These main components have been defined in detail:

(1) Learning objectives indicate the expected level of student understanding and performance. They tell students what they have to do, how they should do it, and how they will be assessed. Both course overview and learning objectives involve intended learning outcomes.
(2) Teaching/learning activities are a set of learning processes that the students have to complete by themselves to achieve a given course’s intended learning outcomes. In BL, activities include both online and face-to-face activities where students are able to engage in collaborations and social interactions (Hadwin and Oshige, 2011; Ellis et al., 2021). The interactive learning activities are chosen to best support course objectives and students’ learning outcomes (Clark and Post, 2021). Examples of activities in BL include: group problem-solving, discussion with peers/teachers, peer instruction, answering clicker questions or in-class polls (Matsushita, 2017).
(3) Assessment tasks are tools to determine students’ achievements based on evidence. In BL, assessments can be conducted either online or in-class. Examples of assessments in BL include: online quizzes, group projects, field-work notes, individual assignments.
(4) Besides, based on the definition of BL and the integration of information technology improvement in recent years, online resources and technological support have become essential components of BL courses (Darling-Aduana and Heinrich, 2018; Turvey and Pachler, 2020). On a similar note, Ellis and Goodyear (2016) and Laurillard (2013) emphasized the role of technical devices in BL, whilst Zawacki-Richter (2009) regarded online resources and technological support as central to achieving BL course requirements. In addition, Liu (2021) suggested that a BL model should include teaching objectives, operating procedures, teaching evaluation, and teaching resources before class, during class, and after class, respectively. With this in mind, the present study integrates both essential curriculum components in the face-to-face course and information technology into the teaching and learning aspects of the BL course.

### 2.3. BL effectiveness and SLOs

Many researchers have demonstrated the benefits of BL approach on SLOs because of the importance of BL in improving teaching methods and better reflecting the improvement of the learner skills, talents, and interest in learning. Garrison and Kanuka (2004) reported increased completion rates as a result of BL application. Similar results were agreed upon by other researchers. Kenney and Newcombe (2011) and Demirkol and Kazu (2014) conducted comparisons and found that students in BL environments had higher average scores than those in non-BL environments. Alsalhi et al. (2021) utilized a quasi-experimental study at Ajman University (*n* = 268) and indicated that the use of BL has a positive effect on students’ academic success in a statistics course. “BL helps to balance a classroom that contains students with different readiness, motivation, and skills to learn. Moreover, BL deviates from traditional teaching and memorizing of students” (Alsalhi et al., 2021, p. 253). No statistical significant difference was found among students based on the variables of the university they attended.

However, researchers also showed that BL approach may not be applicable to all learners or improve their learning outcomes. Oxford Group (2013) reported that about 16% of learners had negative attitudes toward BL, while 26% of learners chose not to complete BL. Kintu et al. (2017) examined the relationship between student characteristics, BL design, and learning outcomes and indicated that BL design is beneficial to raise student satisfaction (*n* = 238). The study also found that BL predicted learning outcomes for learners with high self-regulation skills. Similar results were reported by Siemens (2005) who indicated that students who have higher learner interactions resulted in higher satisfaction and learning outcomes. Hara (2000) identified ambiguous course design and potential technical difficulties as major barriers in BL practice, which led to dissatisfied learning outcomes. Clark and Post (2021) utilized a hybrid study in higher education to explore the effectiveness of different instructional approaches (face-to-face, eLearning, and BL) and indicated that the individual student valued active learning in both face-to-face classes and eLearning classes. Moreover, having an eLearning experience prior to face-to-face classes is beneficial for students to perform well on the assessment. However, the study noted that students who took face-to-face courses were positively associated with their final grades.

### 2.4. Research questions and hypotheses

To fill in the gaps, the research questions and hypotheses were raised in the present research as follows:

*RQ1*: What components (among course overview, course objectives, assessments, activities, course resources, and technology support) contribute to the measurement?

*RQ2*: Is there an association between BL effectiveness and SLOs in higher education?

*H1*: All components (among course overview, course objectives, assessments, class activities, course resources, and technology support) contribute to the BL course model.

*H2*: There is an association between BL effectiveness and SLOs.

## 3. Methodology

The study employed a non-experimental, correlational design and used survey responses from undergraduates to address the research questions. Specifically, a higher education institution in Shanghai with a specialization in teacher education was studied. The present study was a part of an instructional initiative project at this institution designed to identify students’ perceptions of the effectiveness of BL and explore the possible relationships between BL effectiveness and SLOs.

The present research consisted of two stages: Stage I (from March 2021 to July 2021) aimed to develop a measurement for evaluating undergraduates’ BL perceptions through a survey of undergraduates who had experienced BL courses. Stage II (from September 2021 to January 2022) aimed to use the developed measurement to examine whether or not there is an association between BL effectiveness and SLOs.

### 3.1. Instruments

#### 3.1.1. Effectiveness of BL scale (EBLS)

In Stage I, according to Biggs’ theoretical framework and the existing literature, the measurement used in this study was composed of six sub-scales: course overview, learning objectives, assessments, course resources, teaching/learning activities, and technology support. After comparing these criteria, the instrument titled “Blended Learning Evaluation” was derived from Quality Matters Course Design Rubric Standards (QM Rubric) and revised. Following consultation with experienced teaching experts who had experience in BL design and application, the revised QM Rubric can be applied to both the online and face-to-face portions of the course. Table 1 details the modified measurement.

**Table 1:** Items of measurement.

Subscales	Descriptions	Example of items
Course overview (4 items)	Students know how to start the course and understand the course overview that covers the purposes, learning process, and teaching method. Students can find the syllabus, assignments, and deadlines from the course webpage as well.	I can find each class’s topics, assignments, and deadlines on the course website or syllabus.
Learning objectives (4 items)	Students feel that the course objectives are set up from students’ perspectives and are aligned with the learning content, class activities, assignments, and assessments.	I can feel that the course objectives are conveyed completely in each lesson content and listed in the form of learning weeks.
Assessments (4 items)	Students feel that the online/offline assessments in this course are suitable for all-level students and help them measure whether or not they have achieved the learning objectives. The assessments are clearly described with rubrics. Students are given useful feedback on their learning progress.	The online/offline learning assessment (including tests, classroom exercises, presentations, etc.) in the course is diversified and progressive, which is adaptive for the level and type of course.
Course resources (5 items)	Students feel that the course resources are useful, suitable, various, and related to the course objectives and their learning outcomes.	I know that this course’s online/offline teaching materials and resources (including courseware, videos, pictures, cases, reading texts, etc.) have correctly labeled sources, which are diversified and conform to teaching logic.
Teaching/learning activities (4 items)	Students feel they achieve the learning objectives and improve their cooperative learning skills through learning activities (including classroom activities and online activities). During the course learning, students regularly receive learning reminders, course notices, homework feedback, and other information.	Learning activities (including classroom and online) provide me with interactive opportunities and guide my active learning.
Technology support (4 items)	The online learning platform includes academic service information and school technical service information that is related to the course or links to relevant websites (such as user guides, technical FAQs, etc). The online course platform and website are easy to use.	Communication applications such as online course content construction and online forums are functional perfection and convenient to use.

Then, a panel of two experts, two blended course design trainers, and two faculty members in the curriculum and instruction department were asked to evaluate the appropriateness and relevance of each item included in the instrument. Subsequently, a group of 10 sophomores and senior students were asked to check how the questions are read and understood and accordingly give feedback. Based on their comments and the suggestions from the panel, a few minor changes were made and content validity was again evaluated by the panel of experts prior to the administration of the instrument.

Finally, the EBLS that modified from Quality Matters Course Design Rubric Standards was determined as the initial scale that was preparing for the construct validity and reliability checks. The EBLS composed of six sub-scales (25 items in total): course overview (4 items), course objectives (4 items), assessments (4 items), course resources (5 items), in-class and online activities (4 items), and technology support (4 items). The measurement applied a 5-point Likert Scale (1-Strongly Disagree, 2-Disagree, 3-So-so, 4-Agree, and 5-Strongly Agree).

#### 3.1.2. Student learning outcomes

In Stage II, the course marks from the *Curriculum and Instruction Theorem* module were used as an indicator of students’ learning outcomes. Multiple regression analysis was utilized via SPSS 25.0 to perform data analysis.

In the second stage of the study, the students’ course marks from the *Curriculum and Instruction Theorem* module were used as an indicator of students’ learning outcomes. This curriculum was a semester-long mandatory course for 91 sophomores which ran for 16 weeks from September 2021 to January 2022. It aimed to develop the students’ knowledge of in-depth disciplinary and academic content but also skills pertaining to cooperation, technology, inquiry, discussion, presentation, and reflection. The course was designed as a synchronous BL curriculum, in which students all had both face-to-face and technologically-mediated interactions (see Table 2). Each week, there were 1.5 h of face-to-face learning that combined lectures, tutorials, and fieldwork. The lectures covered teaching key concepts with examples and non-examples and connected teaching theories to practical issues. Meanwhile, the tutorials provided opportunities for students to collaborate with peers or in groups. The fieldwork offered opportunities for students to observe real classes and interview cooperative teachers or students in local elementary schools. Technologically-mediated interactions supported by the Learning Management System (LMS) provided supplementary learning resources, reading materials, relative videos, cases, assessment and other resources from the Internet. Students were required to complete online quizzes, assignments, projects, and discussions as well on LMS.

**Table 2:** Weekly blended learning design mode.

Weekly BL phase	Pre-f2f session (On LMS)	f2f session	Post-f2f session (On LMS/during office hour)
Hour	0.5 h	1 h	0.5 h
Objectives	Students are able to perceive basic content-based knowledge through readings and video tapes. Students are able to post their misconceptions.	Students are able to summarize main ideas of content-based knowledge. Students are able to analyze scenarios by using the related theorems. Students are able to apply curriculum and instruction theorems in the real world situations.	Students are able to reflect their weekly learning. Students are able to evaluate other groups’/individual’s projects by using rubrics. Students are able to continually exchange their perspectives on LMS discussion platform if available.
Resources	Reading materials, quizzes	Mini lecture, tasks, cases, projects	Rubrics, discussions
Teacher’s behaviors	Lecturer announces weekly tasks to students through LMS. Lecturer posts reading materials, video tapes, and preview quizzes through LMS.	Lecturer discusses misconceptions with students. Lecturer organizes small group activities to explore and exchange perspectives about preview. Lecturer organizes case study/PBL in groups.	Lecturer guides student reflect learning by posting discussion prompts on LMS. Lecturer organizes peer-reviewing/peer-grading by using rubrics. Lecturer provides office hours for students if they need. Lecturer evaluates individual’s learning outcomes.
Students’ behaviors	Students read notes and perform tasks (i.e.: watching video tapes, readings, and completing pre-view quizzes). Students post questions about misconceptions on LMS discussion platform.	Students discuss misconceptions with lecture and peers. Students participate small group activities to express their understanding about preview. Students analyze scenarios by using the related concepts in groups. Students discuss and present their ways about solving real classroom problems with related theorems in groups.	Students reflect their learning on LMS. Students do peer-reviewing/peer-grading by using rubrics. Students continually post/exchange their perspectives on LMS or explore their ideas with the lecturer during the office hours.
Assessments	LMS tasks (videos and readings) Preview quizzes	Class performance Group discussion Presentation with posters or PowerPoints	Reflections Evaluations Discussion (if necessary)

The final marks of the course were derived from both formative and summative assessments. The formative assessments covered attendance and participation, individual assignments (quizzes, reflections, discussions, case studies, and class observation reports) and group projects (lesson plan analysis, mini-instruction, reports). The summative assessment was a paper-based final examination, as required by the college administrators.

### 3.2. Participants

In Stage I of the study, the target population was sophomore and junior undergraduates from different majors at a higher education institution in Shanghai. Detailed demographic information has been reported in the results section of this study. Notably, due to practical constraints, a convenience sample was employed in the present study. As explained by McMillan and Schumacher (2010), although the generalizability of the results is more limited, the findings are nevertheless useful when considering BL effectiveness. Thus, care was taken to gather the demographic background information on the respondents to ensure an accurate description of the participants could be achieved.

In Stage II of the study, the participants were 91 sophomores who took the synchronous BL course, *Curriculum and Instruction Theorem,* in School of Primary Education in the fall of 2021 (September 2021–January 2022).

### 3.3. Data collection procedures, analysis and presentation

Institutional Review Board (IRB) approval was obtained prior to the collection of Stage I and Stage II. In Stage I, the informed IRB-approved Informed Consent Form included a brief introduction to the study purpose, the length of time required to complete the survey, possible risks and benefits, the researcher’s contact information, etc. It also clarified to the potential respondent that the survey was voluntary and anonymous. SurveyMonkey^1^ was used to administer the survey. In Stage II, the informed IRB-approved Informed Consent Form was also provided to participants. LMS was used for data collection.

To address RQ1, the study used the four following steps:

1. An initial measurement was modified and translated from QM rubrics, and the content validity was checked by the authority.
2. Secondly, the reliability of measurement was examined.
3. Exploratory factor analysis (EFA) was conducted to test the construct validity.
4. Confirmatory factor analysis (CFA) was examined to correct for the relationships between the modeling and data. Ultimately, a revised BL measurement was developed with factor loadings and weights. In Stage I, SPSS 26.0 and AMOS 23.0 were utilized to implement factor analysis and structured equation modeling.

To address RQ2, the study followed two steps:

1. Descriptive statistics (mean, standard deviation, minimum rating, and maximum rating) were calculated on the undergraduates’ perspectives on BL effectiveness, as identified by the author.
2. SLOs were regressed on the perceived BL effectiveness. This research question examined whether the overall BL effectiveness was associated with student achievement. In Stage II, SPSS 26.0 was utilized to implement correlations and multiple regressions.

### 3.4. Limitations

Based on the threats to the validity of internal, external, structural, and statistical findings summarized by McMillan and Schumacher (2010), the following limitations of this study are acknowledged. First, since data were self-reported by participants, may have been influenced and the answers they provided may not reflect their true feelings or behaviors. Second, the study used a convenience sample rather than a database consisting of all undergraduates in higher education in Shanghai; therefore, the population external validity was limited to those faculties with response characteristics. Last, although care was taken to generally phrase the research questions in terms of association rather than effects, a limitation of the study is that correlational design limits our ability to draw causal inferences. The results may be suggestive, but further research is needed in order to draw conclusions about BL impacts.

## 4. Results

### 4.1. What factors (among course overview, course objectives, assessments, class activities, course resources, and technology support) contribute to the measurement?

#### 4.1.1. Demographic information in stage I

In Stage I, a survey with 25 items in 6 sub-scales was delivered to undergraduates who had experienced BL in higher education. In total, 295 valid questionnaires were collected in Stage I (from March 2021 to July 2021). Demographic information of the participants were reported as follows: the percentage of male respondents was 27% while the percentage of female respondents was 73%. The majors of respondents included education (51%), literature (22%), computer science (11%), business (10%), arts (5%), and others (1%). All the respondents were single and aged in the range of 19–20 years old.

#### 4.1.2. Reliability analysis

To address RQ1, reliability and EFA were conducted on the questionnaire results. Test reliability refers to “the consistency of measurement – the extent to which the results are similar over different forms of the same instrument or occasions of data collection” (McMillan and Schumacher, 2010, p. 179). To be precise, the study tested internal consistency (Cronbach’s Alpha), composite reliability (CR), and Average of Variance Extracted (AVE) evidence for reliability. According to Table 3, the reliability of the measurement (25 Items) showed the internal reliability for this scale was 0.949 (*N* = 295). The alpha reliability value for each sub-scale is as follows: 0.859, 0.873, 0.877, 0.910, 0.902, and 0.881, respectively. Since the total scale’s alpha value and sub-scales’ alpha values were all greater than 0.70, the reliability of the survey was relatively high and therefore acceptable. Moreover, the AVE of each sub-scale was greater than 0.50, indicating that the reliability and convergence of this measurement were good. In addition, CR values were all greater than 0.80. This indicates that the composite reliability is high. Therefore, this blended course evaluation measurement is deemed reliable.

**Table 3:** Reliability results for the measurement (*N* = 295).

Variable	Item	Corrected item-total correlation	Cronbach’s alpha if item deleted	Cronbach’s alpha	CR	AVE
Course overview	#1	0.703	0.822	0.859	0.859	0.604
	#2	0.695	0.825			
	#3	0.703	0.822			
	#4	0.717	0.816			
Course objectives	#5	0.740	0.833	0.873	0.876	0.638
	#6	0.696	0.853			
	#7	0.701	0.848			
	#8	0.782	0.816			
Assessments	#9	0.704	0.854	0.877	0.878	0.643
	#10	0.726	0.845			
	#11	0.779	0.824			
	#12	0.730	0.844			
Course resources	#13	0.770	0.891	0.910	0.910	0.671
	#14	0.807	0.883			
	#15	0.782	0.888			
	#16	0.743	0.896			
	#17	0.759	0.893			
Activities	#18	0.787	0.871	0.902	0.902	0.697
	#19	0.787	0.871			
	#20	0.782	0.873			
	#21	0.766	0.878			
Technology support	#22	0.715	0.858	0.881	0.882	0.653
	#23	0.725	0.854			
	#24	0.745	0.847			
	#25	0.788	0.829			

#### 4.1.3. Exploratory factor analysis

According to the research design, EFA was then carried out to determine its construct validity by using SPSS 26.0 to identify if some or all factors (among course overview, course objectives, assessments, class activities, course resources, and technology support) perform well in the context of a blended course design. According to Bryant and Arnold (1995), to run EFA, the sample should be at least five times the number of variables. The subjects-to-variables ratio should be 5 or greater. Furthermore, every analysis should be based on “a minimum of 100 observations regardless of the subjects-to-variables ratio” (p. 100). This study included 25 variables, meaning that 300 samples were gathered. The number of samples was more than 12 times greater than the variables. Compared to the criteria proposed by Kaiser and Rice (1974), the KMO of measurement in this study was greater than 0.70 (0.932). This result indicates the sampling is more than adequate. According to Table 4 (showing Bartlett’s Test of Sphericity), the approximate Chi-square of Bartlett’s test of Sphericity is 4124.801 (*p* = 0.000 < 0.001). This shows that the test was likely to be significant. Therefore, EFA could be used to examine the study.

**Table 4:** Bartlett’s test of sphericity.

Kaiser-Meyer-Olkin measure of sampling		0.932
Bartlett’s test of	Approx. Chi-Square	4124.801
	df	300
	Sig.	0.000

EFA refers to “how items are related to each other and how different parts of an instrument are related” (McMillan and Schumacher, 2010, p. 176). Factor analysis (principal component with varimax rotation) analysis was deployed to assess the degree to which 25 blended course design level questions were asked in the “Blended Course Evaluation Survey.” According to the EFA results detailed in Table 5 (Rotated Factor Matrix), the 25 items loaded on six factors with eigenvalues were greater than 1. The results of the rotated factor matrix showed the loadings were all close to or higher than 0.70 (Comrey and Lee, 1992). Therefore, these six factors mapped well to the dimensions and the measurement can be seen to have relatively good construct validity. Hence, to answer RQ1, all of the factors (among course overview, course objectives, assessments, class activities, course resources, and technology support) performed well in the measurement.

**Table 5:** Rotated factor matrix*.

	Component					
Item #	Resources	Activities	Technology	Assessment	Objectives	Overview
#22	**0.818**	0.184	0.172	0.124	0.163	0.127
#23	**0.812**	0.104	0.097	0.170	0.149	0.153
#21	**0.788**	0.071	0.179	0.250	0.114	0.125
#24	**0.755**	0.130	0.197	0.108	0.143	0.193
#25	**0.748**	0.175	0.143	0.218	0.181	0.180
#18	0.144	**0.800**	0.212	0.127	0.186	0.160
#20	0.086	**0.799**	0.195	0.131	0.177	0.186
#19	0.200	**0.778**	0.219	0.219	0.065	0.204
#17	0.197	**0.772**	0.234	0.129	0.201	0.179
#16	0.177	0.127	**0.835**	0.165	0.166	0.086
#15	0.174	0.208	**0.792**	0.152	0.059	0.149
#13	0.178	0.235	**0.760**	0.153	0.098	0.097
#14	0.172	0.259	**0.724**	0.145	0.174	0.182
#11	0.174	0.120	0.128	**0.819**	0.158	0.159
#12	0.176	0.147	0.200	**0.784**	0.122	0.109
#10	0.225	0.156	0.153	**0.765**	0.159	0.104
#9	0.176	0.129	0.122	**0.752**	0.206	0.142
#8	0.148	0.162	0.080	0.273	**0.818**	0.145
#6	0.217	0.162	0.211	0.104	**0.747**	0.166
#5	0.253	0.109	0.144	0.201	**0.734**	0.244
#7	0.115	0.224	0.094	0.164	**0.690**	0.352
#1	0.162	0.188	0.097	0.164	0.190	**0.770**
#2	0.150	0.207	0.071	0.209	0.180	**0.765**
#4	0.242	0.220	0.177	0.043	0.331	**0.684**
#3	0.286	0.153	0.299	0.148	0.213	**0.668**
Eigenvalue	3.787	3.101	3.053	3.041	2.870	2.722
% of Variance	15.148	12.403	12.212	12.165	11.481	10.886
Cumulative %	15.148	27.552	39.764	51.929	63.411	74.297

Extraction Method: Principal Axis Factoring. Rotation Method: Varimax with Kaiser Normalization.

* Rotation converged in 16 iterations. The bold values indicate that load size of the factors are greater than 0.5.

To further address to what extent factors contribute to the measurement, the hypothesized model in the present study was examined, after which the weight of each factor was calculated for educators based on its structural equation modeling. To discern whether the hypothesized model reflects the collected data, AMOS 23.0 was utilized to carry out confirmatory factor analysis. Compared the fit indexes to the criteria in Table 6 Comparison of Fit Indexes for Alternative Models of the Structure of the Blended Course Design Measurement below, the Root Mean Square Error of Approximation (RMSEA) was 0.034, lower than our rule of thumb of 0.05, which would indicate a good model. Additionally, the results of TLI (0.979) and CFI (0.981) were above our target for a good model. Moreover, CMIN/DF was 1.284, lower than 3; GFI was 0.909, greater than 0.8; AGFI was 0.886, greater than 0.8; NFI was 0.922, greater than 0.9; IFI was 0.982, greater than 0.9; and RMR was 0.013 lower than 0.08. Based on these criteria, it appears that the initial model fits the data well. In other words, the initial model can effectively explain and evaluate a blended course design.

**Table 6:** Comparison of fit indexes for alternative models of the structure of the blended learning measurement.

Model	Criteria	Fit index	Judgment
CMIN	–	333.782	–
DF	–	260	–
CMIN/DF	<3	1.284	Good
RMR	<0.08	0.013	Good
GFI	>0.8	0.909	Good
AGFI	>0.8	0.886	Good
NFI	>0.9	0.922	Good
IFI	>0.9	0.982	Good
TLI	>0.9	0.979	Good
CFI	>0.9	0.981	Good
RMSEA	<0.08	0.034	Good

#### 4.1.4. Confirmatory factor analysis

**Figure 1:**
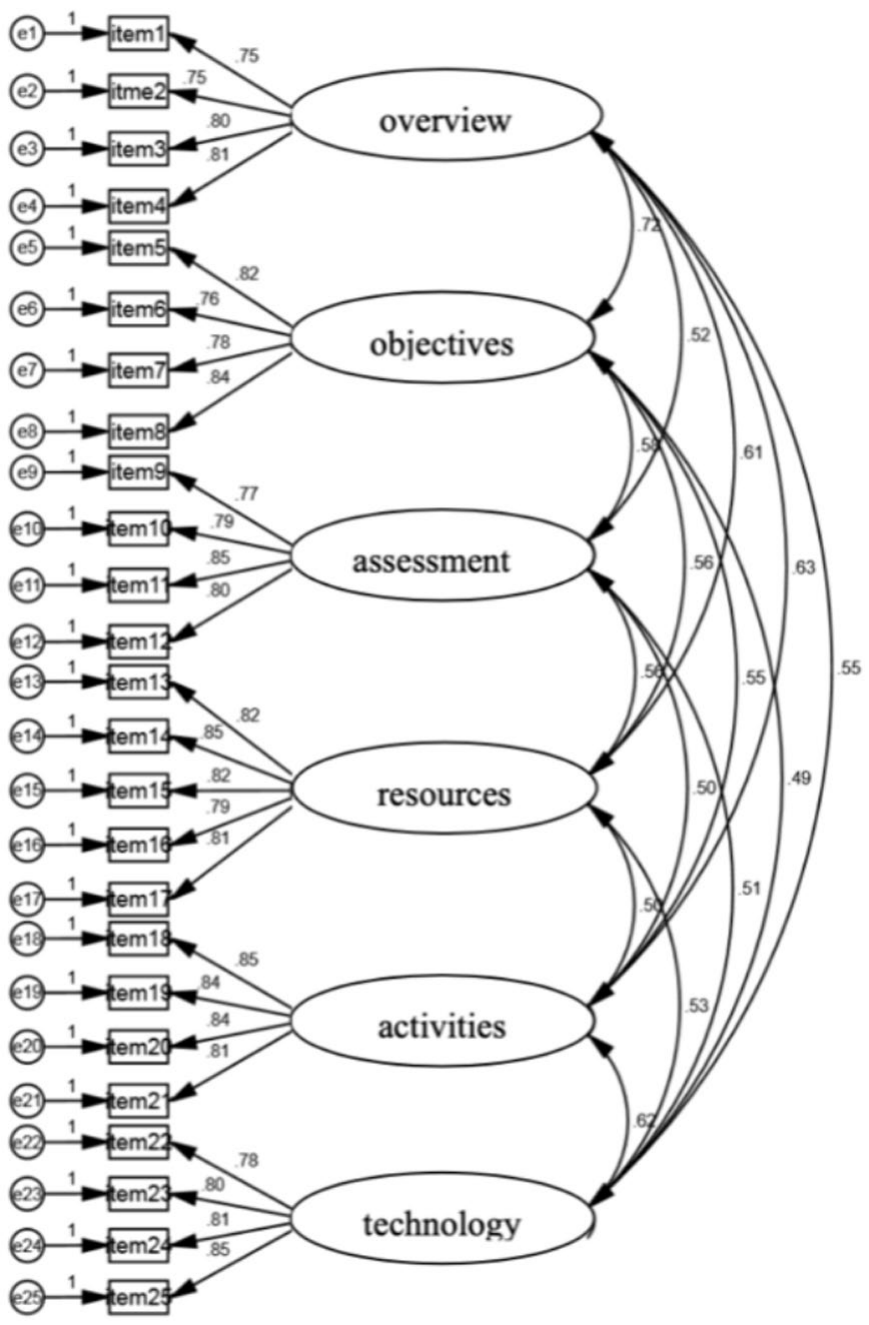
Standardized estimates for the initial blended course design six-factor model.

Focusing on the model itself, CFA was examined to correct the relationships between the modeling and data. Figure 1 shows that most subtests provided relatively strong measures of the appropriate ability or construct. Specifically, one factor was positively correlated to the others. For instance, course overview was positively correlated to course objectives, assessments, course resources, class activities, and technology support. The coefficients of the correlations for the respective factors are as follows: 0.72, 0.52, 0.61, 0.63, 0.55. This means that in any BL, if the course overview rises by 1 point, the other variables will rise by 0.72, 0.52, 0.61, 0.63, 0.55 points, respectively. The results match the statement that “cognitive tests and cognitive factors are positively correlated” (Keith, 2015, p. 335). Additionally, this study tested the discriminant validity of the measurement to ensure that each factor performed differently in the model itself. According to Fornell and Lacker’s (1981) criteria, the square root of AVE value must be greater than the correlation value between the other concepts. The results in Table 7 illustrated that the value of the variables (0.777 which was the lowest) exceeded the correlation value (0.72 which was the greatest). From this, it can be confirmed that the hypothesized model used in the present study had sufficient discriminant validity. Therefore, the hypothesized model in the present study reflected reality well.

**Table 7:** Discriminant validity.

	Overview	Objectives	Assessment	Resources	Activities	Technology
Overview	0.777					
Objectives	0.627**	0.799				
Assessment	0.461**	0.506**	0.802			
Resources	0.535**	0.502**	0.503**	0.819		
Activities	0.553**	0.498**	0.448**	0.450**	0.835	
Technology	0.474**	0.437**	0.455**	0.480**	0.558**	0.808

**Figure 2:**
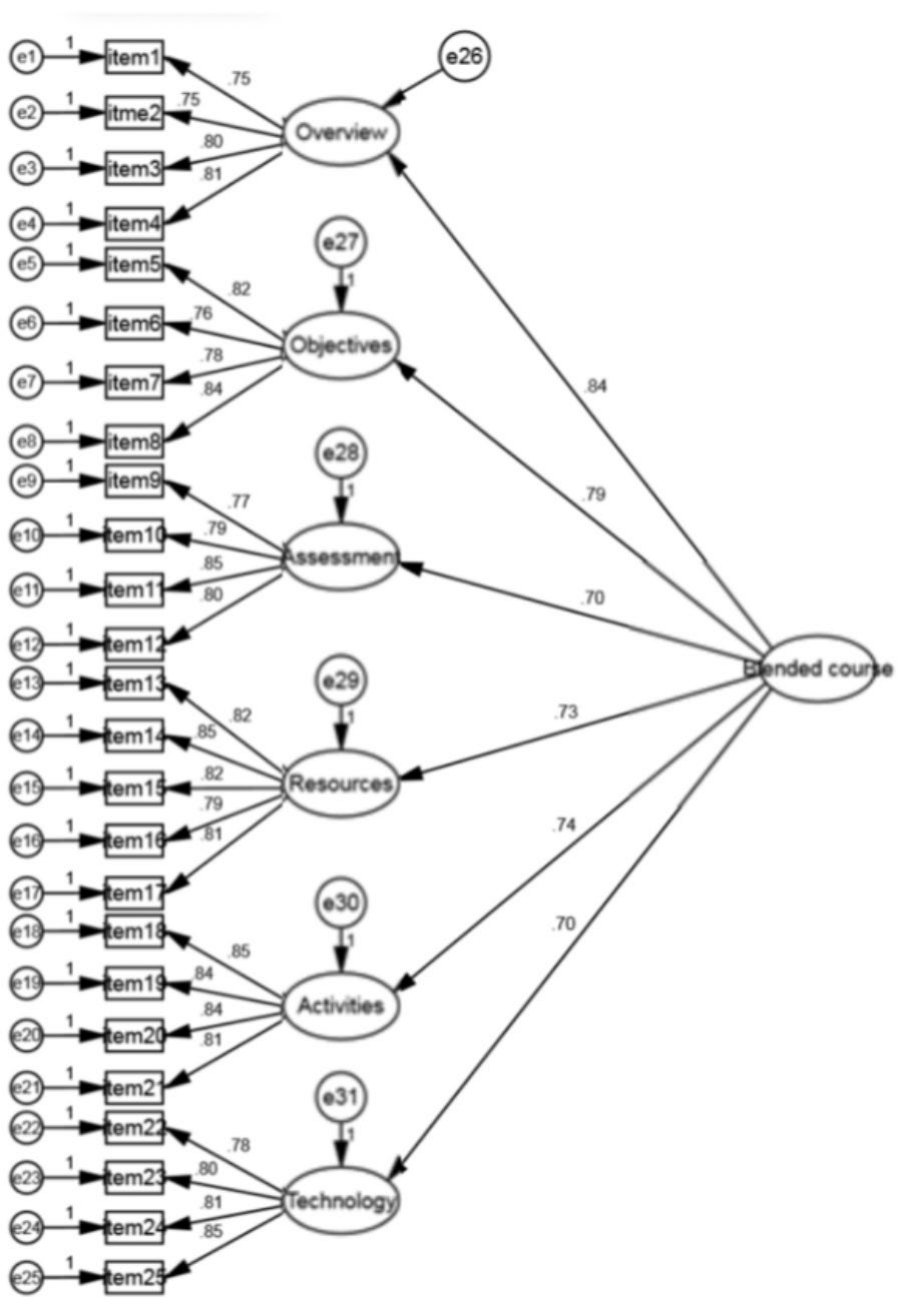
Weight of factors in the present model.

The weight of each factor in the model was further calculated for educators based on structural equation modeling (see Figure 2). For example, the weight of course overview = 0.84/(0.84 + 0.79 + 0.70 + 0.73 + 0.74 + 0.70) = 0.187. Using the same way to calculate the other weighs. The relevant calculations are shown below and the results are shown in Table 8. The total score of a blend course design is calculated as follows: the score of course overview * 0.187 + the score of course objectives * 0.176 + the score of assessment * 0.155 + the score of course resources * 0.162 + the score of class activities * 0.164 + the score of technology support * 0.156. The total grade for this measurement is 100.

**Table 8:** Confirmatory factor loading and weightings.

Variable	Item	Factor loading	Weight of each factor
Course overview	#1	0.754	0.187
	#2	0.748	
	#3	0.795	
	#4	0.809	
Course objectives	#5	0.816	0.176
	#6	0.758	
	#7	0.780	
	#8	0.839	
Assessment	#9	0.767	0.155
	#10	0.795	
	#11	0.846	
	#12	0.797	
Course resources	#13	0.819	0.162
	#14	0.854	
	#15	0.820	
	#16	0.788	
	#17	0.812	
Class activities	#18	0.849	0.164
	#19	0.840	
	#20	0.838	
	#21	0.812	
Technology support	#22	0.776	0.156
	#23	0.801	
	#24	0.807	
	#25	0.846	
Total			1

### 4.2. Is there an association between the effectiveness of blended learning and student learning outcomes?

#### 4.2.1. Demographic information in stage II

In Stage II, there were 91 respondents collected through LMS. The percentage of male respondents was 16% while the percentage of female respondents was 84%. All the respondents in Stage II took the synchronous BL course, *Curriculum and Instruction Theorem,* in School of Primary Education in Fall 2021 (September 2021–January 2022).

#### 4.2.2. Descriptive statistics

In answering RQ2, the descriptive statistics were reported in Table 9 for the undergraduates’ perspectives on BL effectiveness and SLOs. Higher scores for this measure of BL effectiveness indicate undergraduates perceive BL as more effective, with responses of 1 for “Strongly Disagree” to 4 for “Strongly Agree.” The results revealed that the six elements of BL effectiveness had an overall mean of 93.65 (corresponding to an item average of 3.74, which corresponds to “agree”). The scores of each sub-scale are very similar and, again, correspond to undergraduates reporting that they “agree” with the efficacy of BL with respect to course overview, course objectives, assessment, course resources, class activities, and technology support. Table 8 also provided information about overall SLOs. Specifically, it illustrated that students’ learning outcomes (final marks composed of formative assessments and summertime assessments) in BL had an overall mean of 80.65. The maximum and minimum scores were 93.00 and 60.00, respectively.

**Table 9:** Descriptive statistics for the overall scores and sub scales of the measures of blended learning effectiveness and student achievement (*N* = 91).

	Number of items	Cronbach’s Alpha	*M*	SD	Min	Max	Average per item
Blended learning effectiveness (Overall)	25	0.814	93.65	9.19	59.00	100.00	3.74
Student achievement (Overall)	–	–	80.65	7.21	60.00	93.00	–
Course overview	4	0.885	14.90	1.74	6.00	16.00	3.72
Course objectives	4	0.871	15.01	1.57	9.00	16.00	3.75
Assessment	4	0.913	15.05	1.67	8.00	16.00	3.76
Course resources	5	0.885	18.69	1.98	10.00	20.00	3.73
Class activities	4	0.906	15.19	1.46	10.00	16.00	3.79
Technology support	4	0.901	14.79	1.79	9.00	16.00	3.69

#### 4.2.3. Regressions between BL effectiveness and SLOs

To further address the relationship between BL effectiveness and SLOs, SLOs was regressed on the perceived BL effectiveness. This research question examined whether the overall BL effectiveness was associated with student achievement. Additionally, Pearson correlations (shown in Table 10) between key variables were calculated. The results showed that the overall score of BL effectiveness was significantly correlated with student achievement (*r* = 0.716, *p* < 0.01).

**Table 10:** Descriptive statistics and Pearson correlations between key variables in the regression models.

Variables	Correlations						
	2	3	4	5	6	7	8
1. Course overview	0.826**	0.754**	0.849**	0.683**	0.616**	0.882**	0.618**
2. Course objectives	–	0.796**	0.813**	0.782**	0.747**	0.920**	0.660**
3. Assessment	–	–	0.808**	0.821**	0.712**	0.907**	0.642**
4. Course resources	–	–	-	0.776**	0.774**	0.939**	0.667**
5. Class activities	-	-	-	–	0.701**	0.878**	0.615**
6. Technology support	–	–	–	–	–	0.850**	0.646**
7. Blended learning effectiveness (Overall)	–	–	–	–	–	–	0.716**
8. Student achievement (Overall)	–	–	–	–	–	–	–

*p < 0.05; **p < 0.01.

Table 11 shows the results for the regression of total student academic performance on the overall BL effectiveness scores across six components (course overview, course objectives, assessment, course resources, class activities, and technology support). Notably, the full model was statistically significant. Directly addressing RQ2, undergraduates reported that regarding BL effectiveness explained 51.3% of the additional variance, *F*(1, 89) = 93.843, *p* < 0.001, ΔR^2^ = 0.508. Moreover, it was statistically significant and considered to have a large effect. Accordingly, when the perception of BL effectiveness increased by a value of one point, the student’s academic performance would increase by 0.563 (*b* = 0.563, *p* < 0.001). Thus, to answer the final research question, there is a positive correlation between the effectiveness of BL and student learning achievement.

**Table 11:** Summary of simultaneous multiple linear regression results predicting student achievement from perceptions of the blended learning effectiveness.

	*b*	SE_b_	*β*	*t*	*p*
Predictor Variables					
Blended learning effectiveness (Overall)	0.563	0.058	0.716	9.687	0.000**

*p < 0.05; **p < 0.01. *R* = 0.716, *R*^2^ = 0.513, *F*(1, 89) = 93.843, *p* < 0.001.

## 5. Conclusion and discussion

BL is a combination of face-to-face interactions and online learning, where the instructor manages students in a technological learning environment. In the post-pandemic era, BL courses are widely used and accepted by educators, students, and universities. However, the validity of BL remains controversial. The lack of an accurate BL scale was one of the big concerns. The study developed a measurement to evaluate BL for undergraduates and investigated the relationship between the effectiveness of BL and SLOs. Biggs’ 1999) constructive alignment, including factors like course overview, learning objectives, teaching/learning activities, and assessment, was utilized as the primary theoretical framework for conceptualizing the scale. Later, related literature indicated the importance of adding technology and resources as essential components. Therefore, a scale was developed with six subscales.

RQ1 explored the essential components of BL. Stage I recruited 295 undergraduates from different majors at a university in Shanghai. Hypothetical measurements that include 6 sub-scales (25 items in total) were examined. Construct validity was examined with EFA and CFA. As a result, a 6-factor 5-point Likert-type scale of BL effectiveness made up of 25 times was developed. The total variance regarding the six factors of this scale was calculated as 68.4%. The internal consistency reliability coefficient (Cronbach’s alpha) for the total scale was calculated to be 0.949. The alpha reliability values for each sub-scale were as follows: 0.859, 0.873, 0.877, 0.910, 0.902, and 0.881, respectively. The results of the study demonstrated that the hypothesized factors (course overview, course objectives, assessments, class activities, course resources, and technology support) mainly proposed by Biggs (1999) are aligned as a unified system in BL. Furthermore, the results reflect the real concerns of students as they experience BL in higher education However, the participants in the present study were selected from among students enrolled in BL at the university. The characteristics of these samples were as limited as the responders. In future research, a larger scale including undergraduates in other universities may be recruited to test the validity.

RQ2 examined the association between BL validity and SLOs. In Stage II, the study recruited 91 students who participated in a synchronous BL course at the College of Education. The results demonstrated a positive relationship between the effectiveness of BL and SLOs: the more effective that undergraduates perceived BL, the better their SLOs. It supported the results of the previous literature (Demirkol and Kazu, 2014; Alsalhi et al., 2021). Moreover, the descriptive analysis provided additional findings for educators when designing and implementing BL for undergraduates. First, undergraduates expect a clear class overview about how to start the course, how to learn through the course, and how to evaluate their learning outcomes. A clear syllabus with detailed explanations should be prepared and distributed at the outset of BL. Second, undergraduates pay attention to curriculum objectives and continuously compare their work as they progress through the course to see if it helps them achieve those objectives; on this basis, outlining the objectives at the beginning of chapter learning and showing expected learning outcomes (such as rubrics) are recommended. Finally, undergraduates enjoy rich social interactions in both face-to-face activities and online interactions, therefore, a variety of classroom activities for different levels of students is recommended. In future study, more detailed analyses could be considered. For example, it would be valuable to explore the indirect effect of the effectiveness of BL on SLOS. Besides, qualitative research could be conducted to identify the underlying reasons why BL affects SLOs.

## Data availability statement

The datasets presented in this article are not readily available because the datasets generated for this study are not publicly available due to the permissions gained from the target group. Requests to access the datasets should be directed to XH, 158499958@qq.com.

## Ethics statement

The studies involving human participants were reviewed and approved by the Shanghai Normal University Tianhua College. The patients/participants provided their written informed consent to participate in this study.

## Author contributions

XH: drafting the manuscript, data analysis and perform the analysis, and funding acquisition. JF: theoretical framework, and methodology. BW: supervision. YC: data collection and curation. YW: reviewing and editing. The author confirms being the sole contributor of this work and has approved it for publication.

## Funding

This research was supported by the Chinese Association for Non-Government Education (grant number: CANFZG22268) and Shanghai High Education Novice Teacher Training Funding Plan (grant number: ZZ202231022).

## Conflict of interest

The author declares that the research was conducted in the absence of any commercial or financial relationships that could be construed as a potential conflict of interest.

## Publisher’s note

All claims expressed in this article are solely those of the authors and do not necessarily represent those of their affiliated organizations, or those of the publisher, the editors and the reviewers. Any product that may be evaluated in this article, or claim that may be made by its manufacturer, is not guaranteed or endorsed by the publisher.

## Footnotes

^1^ www.surveymonkey.com
